# Discrepancies as to the Number of the Insured

**Published:** 1915-01-30

**Authors:** 


### London Panel Committee.

DISCREPANCIES AS TO THE NUMBER OF THE INSURED.

The London Panel Committee on Tuesday last considered as a matter of urgency an intimation by the Medical Benefit Sub-Committee of the Insurance Committee, that, owing to discrepancies between the number of insured persons on the list in the office of the Insurance Committee and the number returned by practitioners, it would not be possible to make two payments on account of 10d. a quarter to practitioners, but that the payments must be made at the rate of 9d. pending adjustment of the lists.

Dr. H. H. Mills, chairman of the Medical Benefit Sub-Committee of the Insurance Committee, said that the discrepancies were partly due to the withdrawal of a large number of insured persons who had joined the Forces, and partly to the "appalling condition of uncertainty" of the Insurance Committee's register. The first payment for 1915 would be due early in February, and if the Panel Committee did not agree to the proposal of the Insurance Committee delay in disbursing the money would probably ensue.

Protests were made in subsequent speeches at a departure from the mode of payment that had been agreed upon; but, on the other hand, it was urged that the money would be received by practitioners eventually, and that if it were paid upon incorrect lists some practitioners would receive either more or less than the amount rightly due to them. It was stated that the names of many thousands of persons appeared on more than one doctor's list.

Text of the Resolution.

The following resolution was ultimately carried:—

That the Panel Committee concurs in the proposal of the Insurance Committee to pay each practitioner on the panel during the first quarter of 1915 two payments on account of 9d. each in respect of each insured person on his list; and, in so far as the reduction in the practitioner's credits is due to the unsatisfactory state of the medical register and the register of insured persons of the London Insurance Committee, formally records its dissatisfaction with the efficiency of the Committee staff at 5 Chancery Lane, and hopes the Insurance Committee will take stringent immediate steps to provide an efficient administrative service.

Alleged Excessive Prescribing.

The Pharmacy Sub-Committee reported having considered further cases in which the Pharmaceutical Committee made representations that the cost of drugs and appliances ordered was apparently in excess of reasonable requirements. In eight cases the Sub-Committee found that the prescriptions were not excessive, in nineteen cases it found the evidence insufficient for a decision to be reached, and in eighteen cases excessive prescribing, either generally or in specific instances, was found to be proved.

As to a case which had been referred back by the Panel Committee for further consideration, the Pharmacy Sub-Committee adhered to its opinion that the prescribing was not excessive. In this case—rheumatism—phylacogen had been ordered at the cost of £7, acne vaccine at a cost of £5, and Kepler's ext. malt c. ol. morrh. at a cost of £5 5s.

In answer to criticism, it was stated that the practitioner justified the prescription of the vaccines on the ground that they had proved effective in stubborn cases. Dr. C. W. Hogarth, chairman of the Sub-Committee, warned the Committee against interfering with a practitioner's right to use his own judgment as to what was best for his patient. The Committee should only deal with cases in which careless or thoughtless prescribing had apparently taken place. It was also urged that doctors who, in special cases, ordered an expensive drug, did not make so heavy a call upon the Drug Fund as practitioners who were continually careless in regard to dosage. Some speeches by the Chancellor of the Exchequer—notably one in which he described the practitioner under the Insurance Act as being entitled to order medicines for a servant girl precisely as for a duchess—were quoted with great effect.

The Committee decided to accept the Sub-Committee's view that the case was not one of excessive prescribing.

General Decisions.

It was decided to establish a fund to meet expenses which could not be regarded as "administrative expenses" within the meaning of the Act of 1913, under which Panel Committees were established, and to invite contributions from practitioners on the panel.

The action of a Sub-Committee in inviting applications from medical practitioners willing to act as deputies to practitioners on the panel during temporary absences was authorised.

A section was appointed to consider the adequacy of the medical service in London, and the powers and duties of the Panel Committee in connection with the development of the service.

The Committee commended to the notice of practitioners the Report of the Departmental Committee on Sickness Benefit Claims, and endorsed the suggestion therein that further consideration should be given to the scope of medical benefit.

